# Design and Analysis of a General Relay-Node Selection Mechanism on Intersection in Vehicular Networks

**DOI:** 10.3390/s18124251

**Published:** 2018-12-03

**Authors:** Dun Cao, Bin Zheng, Jin Wang, Baofeng Ji, Chunhai Feng

**Affiliations:** 1 School of Computer and Communication Engineering, Changsha University of Science and Technology, Changsha 410114, China; caodun@csust.edu.cn (D.C.); zhengbin@csust.edu.cn (B.Z.); 2 Hunan Provincial Key Laboratory of Intelligent Processing of Big Data on Transportation, Changsha 410114, China; 3 College of Information Engineering, Henan University of Science and Technology, Luoyang 471000, China; baofengji@haust.edu.cn; 4 Department of Computer Science and Engineering, University of Texas at Arlington, Arlington, TX 76019, USA; chunhai.feng@mavs.uta.edu

**Keywords:** performance analysis, relay-node selection, general intersection, vehicular networks

## Abstract

Employment of a relay node can extend the coverage of a message in vehicular networks (VNET). In addition, the prior information regarding the road structure, which determines the structure of VNET, can benefit relay-node selection. However, the non-line-of-sight (NLOS) communication in the intersection scenarios and diverse shapes for the intersection hamper the design of a general relay-node selection on intersection. To resolve this problem, in this paper, we build a model to describe the general intersection, and propose a general relay-node selection method on intersection. Additionally, based on our mathematical description of the general intersection, the performance models for the general relay-node selection on the intersection are first explored in terms of message dissemination speed and Packet Delivery Ratio (PDR). The simulation results validate these models and indicate the improvement of our proposal, especially in heavy traffic. The improvement includes, at the high density of 3.0025 vehicles/m, the huge gain of up to 23.35% in terms of message dissemination speed than that of other compared methods and PDR of over 90%.

## 1. Introduction

In vehicular network (VNET) [1,2], the information about the road, traffic and environment can be shared among vehicles, pedestrians and networks to improve the efficiency and safety of transportation via the technology of both vehicular ad hoc networks (VANET) [3,4,5,6,7,8] and Long-term Evolution enhance Vehicle-to-Everything (LTE eV2X) [9,10,11,12]. Since the communication range of the node is limited, the relay node should be employed to deliver the message that carries this information to a wider area. How to select the relay node to ensure efficient and stable message dissemination, while avoiding the typical problems in the ad hoc network such as the message storm and the hidden node problem, is an interesting issue.

Different from the node in traditional ad hoc networks, the nodes (e.g., vehicles) in VNET are restricted on the road, then the trajectory of nodes can be predicted, and the node distribution can be attained, aided bythe road topology. Additional information on the road can facilitate the relay-node selection. Hence, in the relay-node selection methods, the geography-based method presents an improved performance. Moreover, the popularization of the positioning system and Geographic Information System (GIS) service makes the access to the local road information no longer a limited factor.

The existing relay-node selection methods based on geography information focus on the design of a straight road and the crossroad. However, these methods present a performance degradation in dense networks or sparse networks. In our earlier work [13], an exponent-based partitioning broadcast protocol (EPBP) is proposed. EPBP performs a significant improvement under high node density conditions in terms of message dissemination speed and packet delivery ratio (PDR). Based on exponent partition, we explore a robust relay-node selection in [14], which presents an acceptable performance in the adverse scenario and a high performance in general scenarios, even in sparse density where EPBP is not expert. In the two methods, the application of the exponent partition is just discussed on the straight road.

Aiming at the intersection scenario, this paper develops a stable relay-node selection on a general intersection. The contributions of this paper are threefold: (1) building a model to describe the general intersection with $N_{b}$ branches and any angle between adjacent branches; (2) exploring a general relay-node selection method on intersection based on the exponent partition, which presents a significantly improved and stable performance; (3) constructing the performance analysis of our proposal in terms of message dissemination speed and PDR for the evaluation and future optimization.

The remainder of this paper is organized as follows. In Section 2, the related works are described briefly. After presenting a mathematical description for a general intersection, an exponent-partition-based relay-node selection method on the general intersection is discussed in Section 3. Additionally, the analytic models for the proposed method are developed in Section 4. In addition, Section 5 validates these models and evaluates the improvement of the proposed method by simulation. Finally, our work is summarized.

## 2. Related Work

In the traditional relay-node selection methods for VNET, the information of speed, acceleration, and location of neighbors, which is beneficial to the selection, is enclosed in a periodical beacon, e.g., the method in the known Greedy Perimeter Stateless Routing (GPSR) [15] and its novel methods. However, the interval of two successive beacons (100 ms) makes this information sacrifice its real-time features. The position-based multi-hop broadcast protocol (PMBP) [16] and the trinary partitioned black-burst-based broadcast protocol (3P3B) [17] take advantage of the information of local road structure (the straight road) to select the relay node without the prior knowledge of the neighbor location. However, in Ref. [18], the intersection is considered to develop the urban multi-hop broadcast protocol (UMB) and ad hoc multi-hop broadcast (AMB), as well as the binary-partition-assisted broadcast protocol (BPAB) in Ref. [19]. Based on the road structure, the above four methods partition the communication range into multi-segments and select the node in the final empty segment as the relay node. Consequently, these methods achieved a limited partition latency and relatively small contention latency. However, in the heavy or sparse traffic, these methods would deteriorate seriously.

Meanwhile, there are many existing works to model and analyze the performance of the relay-node selection [20,21,22,23]. However, efforts are still limited in the case of the typical crossroad and straight road. The literature [24] and [25] study the impact of the obstacles in the intersection and propose the network-coded intersection relaying and the selective intersection relaying to improve the performance in terms of PDR, respectively. More efforts need to be made in the study to select the node in realistic scenarios, not only a straight road and crossroad but also an atypical intersection and curved road.

**Figure 1:**
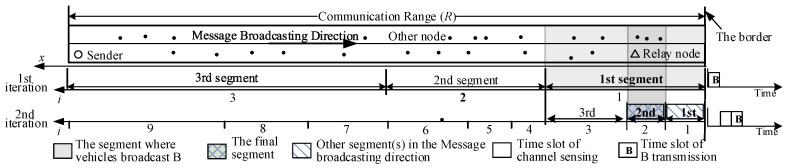
Example of the exponent-based partition, $\left(N_{\mathrm{iter}},N_{\mathrm{part}}\right)=\left(2,3\right)$.

In our earlier work [13], an exponent-based partition method was proposed to select the relay node in a straight road. With the aid of the black burst (BB) (i.e., a channel-jamming signal), the communication range (*R*) of the sender is partitioned into $N_{\mathrm{part}}$ segments with $N_{\mathrm{iter}}$ iteration. Figure 1 shows an example with $\left(N_{\mathrm{iter}},N_{\mathrm{part}}\right)=\left(2,3\right)$. In each iteration, the segment nearer to the border has a smaller width. The width of the *i*-th segment in the *j*-th iteration can be given as

(1) $$W_{\mathrm{seg}}\left(j,i\right)=\frac{1}{A}\left[{\left(1+A\right)}^{\frac{\left(\left(i-1\right)\;\mathrm{mod}\;N_{\mathrm{part}}\right)+1}{N_{\mathrm{part}}}}-{\left(1+A\right)}^{{}^{\frac{\left(i-1\right)\;\mathrm{mod}\;N_{\mathrm{part}}}{N_{\mathrm{part}}}}}\right]W_{\mathrm{seg}}\left(j-1,\left\lceil \frac{i}{N_{\mathrm{part}}}\right\rceil \right)$$

where *A* is a compression coefficient, and ⌈•⌉ denotes the ceiling function. In our robust method [14], based on the closed-form expression of the performance in terms of the message dissemination speed, the optimal parameters of $N_{\mathrm{iter}}$, $N_{\mathrm{part}}$, $N_{A}$ for EPBP are given. Moreover, a mini-black-burst-assisted mechanism (mini-BBM) is developed to reduce the partition latency in the low node density. The robust method performs a stable performance on the straight road. In this paper, the optimization of the exponent partition and mini-BBM also are adapted to explore a general relay-node selection on the intersection.

For clear exposition, the primary notations throughout this paper are summarized in Table 1.

## 3. Design of a General Relay-Node Selection on Intersection

In this section, we firstly construct a model to describe the general intersection. Based on the model, a general relay-node selection method on intersection is further presented to make the message cover the maximum range on every branch. Finally, the exponent-based partition on intersection is explored to achieve less delay and higher PDR.

### 3.1. Description of a General Intersection

**Figure 2:**
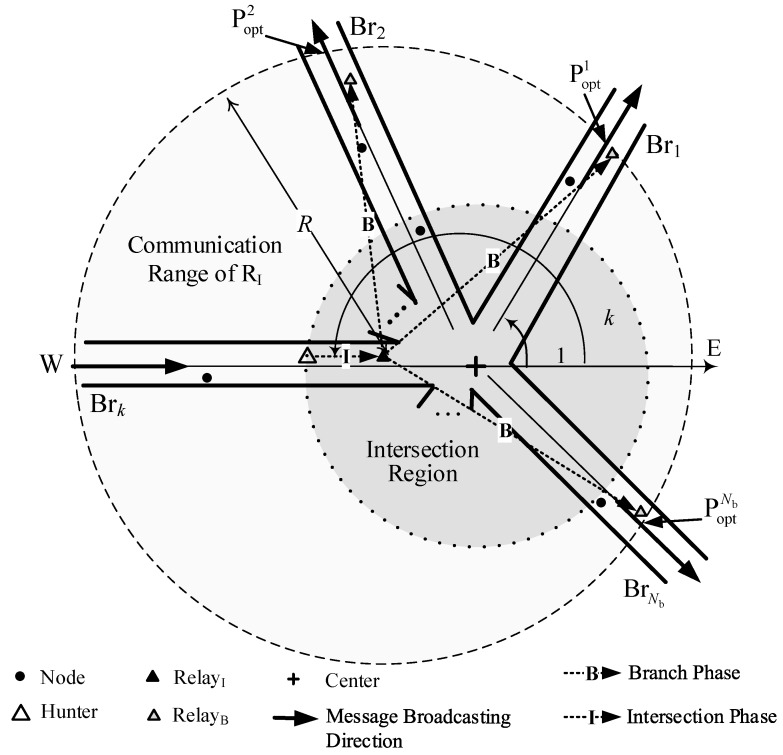
General relay-node selection on intersection.

A general intersection with $N_{b}$ branches is shown in Figure 2. A message comes from one branch and is expected to be delivered to other $N_{b}-1$ branches. Extend the branch so that the message comes from the direction of the message dissemination and build a coordinate system with the origin point at C (the center of the intersection). Branches are marked as ${\mathrm{Br}}_{1}$, ${\mathrm{Br}}_{2}$, ..., ${\mathrm{Br}}_{N_{b}}$ in the counterclockwise direction from the positive axis, and the angles between each branch and the positive axis are denoted as $\theta_{1}$, $\theta_{2}$, ..., $\theta_{N_{b}}$, respectively. In the examples in Figure 2, the message comes from the western branch assumed as ${\mathrm{Br}}_{k}$, and the coordinate axis goes from west to east. The angle between the branch ${\mathrm{Br}}_{k}$ and the positive axis is $\theta_{k}$.

Based on the description of the general intersection, an exponent-partition-based relay-node selection suitable to the realistic intersections is explored in the following subsection, and the analytic performance models are presented in Section 4.

### 3.2. Procedure of the Relay-Node Selection on General Intersection

The objective of our relay-node selection is to deliver the message from ${\mathrm{Br}}_{k}$ to other branches as far and fast as possible. The proposal consists of two phases. The first phase is to find a node closest to C, aiming to cover the largest range in all branches with the selected relay node, referred to as Intersection Phase. In the second phase, referred to as Branch Phase, the farthest nodes in each branch except ${\mathrm{Br}}_{k}$ are selected as relay nodes.

#### 3.2.1. Intersection Phase

Before a message reaches the intersection range, it is assumed to be delivered on a straight road, which is one branch of the intersection. When a selected node finds itself in the Intersection Region, it is assigned a function to select a node closest to C. We refer the node first in the Intersection Region as Hunter (H) and the node closest to C as Relay Node in Intersection Phase (${\mathrm{Relay}}_{I}$). The Intersection Region is defined as a circle region centered at C with radius of *r*, which is shown as the shadow area in Figure 2.

The criterion for the selection of *r* is to cover every possible node which is nearer to C than H. We consider the case that H is located at the border of Intersection Region, i.e., H is *r* away from C. In this case the node superior to H is most unlikely out of the Intersection Region. So *r* should satisfy the inequity as

(2) $$\left\{\begin{array}{l} R^{2}=r^{2}+{\left(l_{B}^{n}\right)}^{2}-2rl_{B}^{n}\cos\left(\theta_{n}-\theta_{m}\right)\\ l_{B}^{n}\geq r \end{array}\right.$$

where *n* is the index of the *n*-th branch (n≠m), and $l_{B}^{n}$ is the coverage of H on the *n*-th branch. Thus, we can derive

(3) r≤R/2

The larger the range in one-hop, the faster the message disseminates. Thus, in our relay-node selection method, r=R/2.

#### 3.2.2. Branch Phase

Having been selected as the relay node, ${\mathrm{Relay}}_{I}$ becomes responsible for initiating the message delivered to all branches except the branch the message comes from. The selection of relay nodes on these branches proceeds independently and simultaneously. Each selection can use different radio channels in VANET or different radio sources in LTE eV2X to transfer the different packets of Request-To-Broadcast/Clear-To-Broadcasting (RTB/CTB) and use different frequency to carry different black bursts on the different branches. In the communication range of ${\mathrm{Relay}}_{I}$, shown as the circle area centered at ${\mathrm{Relay}}_{I}$ in Figure 2, the farthest nodes on the branches except ${\mathrm{Br}}_{k}$ from ${\mathrm{Relay}}_{I}$ are expected to be selected as relay nodes. The relay node in the *n*-th branch is referred to as ${\mathrm{Relay}}_{B}^{n}$. In the following, for simple expression, the relay node on the branch is denoted as ${\mathrm{Relay}}_{B}$.

### 3.3. Exponent-Based Partition on General Intersection

In each phase, we develop the exponent-based partition on the general intersection to select ${\mathrm{Relay}}_{I}$ and ${\mathrm{Relay}}_{B}$ successively. Define the region where the partition is conducted as Partition Rang, referred to as $R_{p}$. From our earlier works [14], it can be derived that the design of a small partition range can reduce the contention latency. Therefore, the exponent-based partition method in each phase is conducted as follows.

#### 3.3.1. Intersection Phase

Since ${\mathrm{Relay}}_{I}$ is expected to be closer to the center than H, the partition range $R_{p}$ is the circle region centered at C with the radius equal to the distance between H and C. In addition, the functionality defined for “Communication Range” and “Border” in Figure 1 is now employed for the $R_{p}$ and C, respectively. The $R_{p}$ is partitioned into $N_{\mathrm{part}}$ circular or ring regions for $N_{\mathrm{iter}}$ iterations to find a thinnest no-empty segment in each branch closest to C, and the thinnest no-empty segment is referred to as final segment. Clearly, the inner segment has higher priority of getting selected over the outer segment. And in each iteration, the segment closer to C has a smaller width. Once the final segment in each branch is determined, nodes located in these segments enter the CTB-contention phase [13]. Finally, one of them is elected as ${\mathrm{Relay}}_{I}$, and the ${\mathrm{Relay}}_{I}$ starts the branch phase.

#### 3.3.2. Branch Phase

In the branch phase, the selection of the node farthest from ${\mathrm{Relay}}_{I}$ means that the node nearest to the junction of each branch and the circle for the communication range of ${\mathrm{Relay}}_{I}$. When ${\mathrm{Relay}}_{B}$ is at the junction, the message progress in the branch phase will be the maximum. We term the junction on the *n*-th branch as the optimal point, $P_{\mathrm{opt}}^{n}$. Since the node nearer to the optimal point than ${\mathrm{Relay}}_{I}$ is expected, the partition range $R_{p}$ is determined as the minimum range among the range from ${\mathrm{Relay}}_{I}$ to the optimal point and the range from C to the optimal point. Consequently, when ${\mathrm{Relay}}_{I}$ in the *m*-th branch, $R_{p}$ on the *n*-th branch can be defined as

(4) $$R_{p}=\left\{\begin{array}{ll} D_{\bar{{\mathrm{Relay}}_{I},P_{\mathrm{opt}}^{n}}}, & \mathrm{when}\;n=m\\ D_{\bar{C,P_{\mathrm{opt}}^{n}}}, & \mathrm{when}\;n\neq m \end{array}\right.$$

where $D_{\bar{A,B}}$ represents the range between the points of A and B. Corresponding $R_{p}$ and $P_{\mathrm{opt}}^{n}$ to “Communication Range” and “Border” in Figure 1, the partition procedure proceeds. After CTB contention, ${\mathrm{Relay}}_{B}$ in each branch is selected. The general relay-node selection on intersection is completed, then the message is delivered to every direction. The mini-Black-Burst-Assisted mechanism in Ref. [14] is also applied in our proposal to alleviate the high partition latency in the adverse scenario [14], in which numerous nodes distribute only in the final segment near the sender, and in the sparse network.

## 4. Modeling and Analysis

Message dissemination speed and PDR are the metrics used widely to validate the efficiency and reliability of vehicular networks. In the scenario of straight road, the selection of relay node in each hop is independent. However, from the description in the above section, the partition range $R_{p}$ for the selection of ${\mathrm{Relay}}_{B}$ is dependent on the location of ${\mathrm{Relay}}_{I}$. It means that the first hop (phase) in the whole procedure decides the latter hop (phase). Thus, we analyze the two metrics in the two hops for our proposed general relay-node selection on intersection. Please note that the two metrics of message dissemination speed and PDR are related to the procedure of the relay-node selection, not the message broadcast.

In the following analysis and the simulation in Section 5, the protocol in [13] is adopted to proceed the message dissemination in one-hop. Message dissemination in this protocol has four phases: Mini-distribute inter-frame space (mini-DIFS) for channel access, exponent-based partition, CTB contention phase to resolve the problem of multiple nodes in the final segment and data transmission. Thus, one-hop delay $T_{d}$ consists of initial latency $T_{\mathrm{init}}$ in mini-DIFS, partition latency $T_{\mathrm{part}}$, contention latency $T_{\mathrm{con}}$ and data transmission latency $T_{\mathrm{data}}$. $T_{d}$ is given as

(5) $$T_{d}=T_{\mathrm{init}}+T_{\mathrm{part}}+T_{\mathrm{con}}+T_{\mathrm{data}}$$

Certainly, other protocol also can be adopted in the message dissemination, and the analysis is similar. In the CTB-contention phase, the exponential back-off timer is applied to alleviate the collision.

### 4.1. Metrics Definition

Since the two phases depend on each other, we redefine Message dissemination speed and PDR to evaluate the performance of our proposal.

#### 4.1.1. Message Dissemination Speed *v*

*v* is the ratio of the message dissemination distance along the road and the delay $\hat{T_{d}}$ in the whole procedure, defined as

(6) $$v=\frac{R_{\max}\beta }{\hat{T_{d}}}$$

where $R_{\max}$ represents the maximal distance of the message dissemination along the roads. β is the message progress which is the message dissemination distance normalized by $R_{\max}$. In addition, $\hat{T_{d}}$ is the delay of the whole procedure. In the scenarios of intersection, $R_{\max}=3R/2$ in the case that H locates at the border of the intersection range, ${\mathrm{Relay}}_{I}$ and ${\mathrm{Relay}}_{B}$ at C and $P_{\mathrm{opt}}$ respectively. β and $\hat{T_{d}}$ are the average value of those corresponding to the message delivered to each branch except ${\mathrm{Br}}_{k}$.

For simple presentation, the message dissemination distance represents the message dissemination distance along the roads in the following subsection.

#### 4.1.2. Packet Delivery Ratio (PDR)

PDR is the percentage of packets received successfully by all relay nodes in branches over the total number of packets sent by Hunter. In this paper, we focus on the selection of the relay node, thus only the failure caused by the collision in CTB contention is considered. This means that the packet fails to be delivered if any relay node on one branch cannot be selected successfully.

In the following subsection, since the exponent-based partitioning method will affect $T_{\mathrm{part}}$ and $T_{\mathrm{con}}$ in each phase, β (the three metrics determinate *v*), and PDR in the whole procedure, we only develop the analytical models for the four metrics of our general relay-node selection on intersection.

### 4.2. Preliminary Analysis

In this subsection, we present the analytical models for these performance metrics in one-hop given the partition range $R_{p}$ and the equivalent node density $\hat{\lambda }$. Based these results, the models of $T_{\mathrm{part}}$, $T_{\mathrm{con}}$, β and PDR for our proposal are explored in the next subsection.

#### 4.2.1. Partitioning Latency $T_{\mathrm{part}}$

Under the assumption that the number of vehicles follows Poisson distribution [16,17,18,19], the probability of the selection of the *i*-th segment in the *j*-th iteration can be derived with a similar procedure in [13] as

(7) $${P^{j,i}}_{\mathrm{seg}\_\mathrm{sel}}\left(\hat{\lambda },R_{p}\right)=e^{-\mu_{\mathrm{seg}\_\mathrm{bro}}^{j,i}\left(\hat{\lambda },R_{p}\right)}\left(1-e^{-\mu_{\mathrm{seg}}^{j,i}\left(\hat{\lambda },R_{p}\right)}\right)$$

where $\mu_{\mathrm{seg}\_\mathrm{bro}}^{j,i}\left(\hat{\lambda },R_{p}\right)$ and $\mu_{\mathrm{seg}}^{j,i}\left(\hat{\lambda },R_{p}\right)$ are the average vehicle numbers in other segments in the message dissemination direction and in the *i*-th segment of the *j*-th iteration, respectively. Note that $\mu_{\mathrm{seg}\_\mathrm{bro}}^{j,i}\left(\hat{\lambda },R_{p}\right)$ and $\mu_{\mathrm{seg}}^{j,i}\left(\hat{\lambda },R_{p}\right)$ are the function of the parameters of $R_{p}$ and $\hat{\lambda }$.

Based on the above results, the average duration $T_{\mathrm{part}}$ spent during the partitioning phase can be obtained as

(8) $$T_{\mathrm{part}}\left(\hat{\lambda },R_{p}\right)=\left(\left(\sum_{j=1}^{N_{\mathrm{iter}}}\sum_{i=1}^{N_{\mathrm{seg}}\left(j\right)}\left(N_{p\_\mathrm{slot}}\left(i\right)P_{\mathrm{seg}\_\mathrm{sel}}^{j,i}\left(\hat{\lambda },R_{p}\right)\right)\right)+1\right)T_{\mathrm{slot}}$$

where $N_{p\_\mathrm{slot}}\left(i\right)$ is the number of time slots spent when the *i*-th segment is selected, $T_{\mathrm{slot}}$ is the duration of a time slot, and $N_{\mathrm{seg}}\left(j\right)={\left(N_{\mathrm{part}}\right)}^{j}$.

#### 4.2.2. Contention Latency $T_{\mathrm{con}}$

With the exponential back-off timer applied, the maximum width of the back-off timer doubles after each collision. Since the contention happens in the nonempty segment, following the similar analysis in [14], the single probabilities of the two cases (success, collision) in the *c*-th contention of the *i*-th final segment can be derived as

(9) $$p_{\mathrm{suc}\_\mathrm{con}}^{i,c}\left(\hat{\lambda },R_{p}\right)=\frac{\mu_{i}\left(\hat{\lambda },R_{p}\right)p_{c}e^{-\mu_{i}\left(\hat{\lambda },R_{p}\right)p_{c}}}{1-e^{-\mu_{i}\left(\hat{\lambda },R_{p}\right)p_{c}}}$$

(10) $$p_{\mathrm{col}\_\mathrm{con}}^{i,c}\left(\hat{\lambda },R_{p}\right)=1-\frac{\mu_{i}\left(\hat{\lambda },R_{p}\right)p_{c}e^{-\mu_{i}\left(\hat{\lambda },R_{p}\right)p_{c}}}{1-e^{-\mu_{i}\left(\hat{\lambda },R_{p}\right)p_{c}}}$$

where $\mu_{i}\left(\hat{\lambda },R_{p}\right)=\hat{\lambda }R_{p}W_{\mathrm{seg}}\left(N_{\mathrm{iter}},i\right)$ is the average number of vehicles in the *i*-th final segment, and $p_{c}=\frac{1}{C_{w}\left(c\right)}$ ($C_{w}\left(c\right)$ is the maximal number of back-off timer in the *c*-th contention). Therefore, the success probability of the whole contention process when the *c*-th contention succeeds in the *k*-th final segment is computed as

(11) $$p_{\mathrm{suc}}^{i,c}\left(\hat{\lambda },R_{p}\right)=p_{\mathrm{suc}\_\mathrm{con}}^{i,c}\left(\hat{\lambda },R_{p}\right)\prod_{l=0}^{c-1}p_{\mathrm{col}\_\mathrm{con}}^{i,l}\left(\hat{\lambda },R_{p}\right)$$

where $p_{\mathrm{col}\_\mathrm{con}}^{k,0}\left(\hat{\lambda },R_{p}\right)=1$ and c=1,2,….

For the similar reasons, the whole contention latency $T_{\mathrm{con}\_\mathrm{seg}}^{i}\left(\hat{\lambda },R_{p}\right)$ of the *i*-th segment can be derived as

(12) $$T_{\mathrm{con}\_\mathrm{seg}}^{i}\left(\hat{\lambda },R_{p}\right)=\sum_{c=1}^{\infty }p_{\mathrm{suc}}^{i,c}\left(\hat{\lambda },R_{p}\right)\left(\sum_{l=0}^{c-1}T_{\mathrm{col}\_\mathrm{si}}\left(l\right)+T_{\mathrm{suc}\_\mathrm{si}}\left(c\right)\right)$$

where $T_{\mathrm{con}\_\mathrm{si}}\left(c\right)$ and $T_{\mathrm{suc}\_\mathrm{si}}\left(c\right)$ are the durations spent in the collision case and the success case in the *c*-th contention, respectively.

It is noteworthy to mention that after some collisions in the case of high vehicle densities, the last success contention cost a little time since multiple nodes contend, and it can be approximated by the success contention duration in the first contention. Finally, the average contention latency in one-hop can be computed as

(13) $$T_{\mathrm{con}}\left(\hat{\lambda },R_{p}\right)=\sum_{i=1}^{N_{\mathrm{seg}}\left(N_{\mathrm{iter}}\right)}T_{\mathrm{con}\_\mathrm{seg}}^{i}\left(\hat{\lambda },R_{p}\right)P_{\mathrm{seg}\_\mathrm{sel}}^{N_{\mathrm{iter}},i}\left(\hat{\lambda },R_{p}\right)$$

#### 4.2.3. One-Hop Message Progress β

One-hop message progress β is the average one-hop transmission distance relative to $R_{p}$ in one hop, which can be calculated as

(14) $$\beta \left(\hat{\lambda },R_{p}\right)=\sum_{i=1}^{N_{\mathrm{seg}}\left(N_{\mathrm{iter}}\right)}M_{i}P_{\mathrm{seg}\_\mathrm{sel}}^{N_{\mathrm{iter}},i}\left(\hat{\lambda },R_{p}\right)$$

where $M_{i}$ is the average message progress if the *i*-th segment is the final segment. Since vehicles distribute in the final segment randomly and each contender has the same probability of being selected, the expected location of the relay node is the middle of the final segment.

#### 4.2.4. PDR

Since in the paper the collision is considered to be the factor for the failure of message delivery, PDR in one hop can be attained as

(15) $$PDR\left(\hat{\lambda },R_{p}\right)=1-\sum_{i=1}^{N_{\mathrm{seg}}\left(N_{\mathrm{iter}}\right)}\left(\left(\prod_{c=1}^{N_{\mathrm{recon}}}p_{\mathrm{col}\_\mathrm{con}}^{i,c}\left(\hat{\lambda },R_{p}\right)\right)P_{\mathrm{seg}\_\mathrm{sel}}^{N_{\mathrm{iter}},i}\left(\hat{\lambda },R_{p}\right)\right)$$

where $N_{\mathrm{recon}}$ is the maximal times for the CTB contention.

### 4.3. Modeling for General Relay-Node Selection on Intersection

To evaluate the selection of the relay-node, we consider the scenarios that there is at least one node in the Intersection Region and in partition range on every branch respectively. In addition, it is assumed that the Hunter locates at the point R/2 from C to analyze the lower bound of the performance since this case has the biggest partition range in the Intersection Phase. In the following, based on the results in the previous subsection, we will first explore the analytic model for the metrics (i.e., $T_{\mathrm{part}}$, $T_{\mathrm{con}}$, β and PDR) in Intersection Phase and Branch Phase, respectively.

#### 4.3.1. Metrics in Intersection Phase

In Intersection Phase, our proposal finds the nearer node to C than H in the circle region centered at C with radius of $D_{\bar{HC}}$. In addition, H is R/2 from C. Therefore, the optimal position $P_{\mathrm{opt}}$ is C, and $R_{p}=R/2$. Moreover, since the circle region covers $N_{b}$ branches, $\hat{\lambda }=N_{b}\lambda$, where λ is the vehicle density in a branch. From (8) and (13), we can obtain the partition latency $T_{\mathrm{part}\_I}$ and the contention latency $T_{\mathrm{con}\_I}$ in Intersection Phase as

(16) $$T_{\mathrm{part}\_I}=T_{\mathrm{part}}\left(N_{b}\lambda ,\frac{R}{2}\right)=\left(\left(\sum_{j=1}^{N_{\mathrm{iter}}}\sum_{i=1}^{N_{\mathrm{seg}}\left(j\right)}\left(N_{p\_\mathrm{slot}}\left(i\right)P_{\mathrm{seg}\_\mathrm{sel}}^{j,i}\left(N_{b}\lambda ,\frac{R}{2}\right)\right)\right)+1\right)T_{\mathrm{slot}}$$

(17) $$T_{\mathrm{con}\_I}=T_{\mathrm{con}}\left(N_{b}\lambda ,\frac{R}{2}\right)=\sum_{i=1}^{N_{\mathrm{seg}}\left(N_{\mathrm{iter}}\right)}T_{\mathrm{con}\_\mathrm{seg}}^{i}\left(N_{b}\lambda ,\frac{R}{2}\right)P_{\mathrm{seg}\_\mathrm{sel}}^{N_{\mathrm{iter}},i}\left(N_{b}\lambda ,\frac{R}{2}\right)$$

and message progress $\beta_{I}$ and $PDR_{I}$ in Intersection Phase from (14) and (15) as

(18) $$\beta_{I}=\beta \left(N_{b}\lambda ,\frac{R}{2}\right)=\sum_{i=1}^{N_{\mathrm{seg}}\left(N_{\mathrm{iter}}\right)}M_{i}P_{\mathrm{seg}\_\mathrm{sel}}^{N_{\mathrm{iter}},i}\left(N_{b}\lambda ,\frac{R}{2}\right)$$

(19) $$PDR_{I}=PDR\left(N_{b}\lambda ,\frac{R}{2}\right)=1-\sum_{i=1}^{N_{\mathrm{seg}}\left(N_{\mathrm{iter}}\right)}\left(\left(\prod_{c=1}^{N_{\mathrm{recon}}}p_{\mathrm{col}\_\mathrm{con}}^{i,c}\left(N_{b}\lambda ,\frac{R}{2}\right)\right)P_{\mathrm{seg}\_\mathrm{sel}}^{N_{\mathrm{iter}},i}\left(N_{b}\lambda ,\frac{R}{2}\right)\right)$$

#### 4.3.2. Metrics in Branch Phase

In Branch Phase, the selection of ${\mathrm{Raley}}_{B}$ is processed on each branch individually and depends on the location of ${\mathrm{Raley}}_{I}$. Hence, $\hat{\lambda }=\lambda$, and the partition range $R_{p\_B}^{n,i,m}$ on the *n*-th branch in Branch Phase when ${\mathrm{Raley}}_{I}$ is in the *i*-th final segment on the *m*-th branch can be achieved as

(20) $$R_{p\_B}^{n,i,m}=\left\{\begin{array}{ll} R, & \mathrm{when}\;n=m\\ l_{C}^{i}\cos\left(\theta_{n}-\theta_{m}\right)+\sqrt{R^{2}-(l_{C}^{i}{)}^{2}\sin^{2}\left(\theta_{n}-\theta_{m}\right)}, & \mathrm{when}\;n\neq m \end{array}\right.$$

where $l_{C}^{i}$ is the distance between ${\mathrm{Raley}}_{I}$ and C when ${\mathrm{Raley}}_{I}$ is in the *i*-th final segment. The derivation of $R_{p\_B}^{n,i,m}$ can be seen in Appendix A.

To avoid confusing, the indexes of the final segments where ${\mathrm{Raley}}_{I}$ and ${\mathrm{Raley}}_{B}$ exist are denoted as $i_{I}$ and $i_{B}$, respectively. Moreover, for simplicity, the notation *i* and $i_{I}$ have the same meaning. From (8) and (13), the partition latency $T_{\mathrm{part}\_B}^{n,i,m}$ and the contention latency $T_{\mathrm{con}\_B}^{n,i,m}$ on the *n*-th branch in Branch Phase when ${\mathrm{Raley}}_{I}$ is in the $i_{I}$-th final segment can be achieved as

(21) $$T_{\mathrm{part}\_B}^{n,i,m}=T_{\mathrm{part}\_B}^{n,i_{I},m}=T_{\mathrm{part}}\left(\lambda ,R_{p\_B}^{n,i_{I},m}\right)=\left(\left(\sum_{j=1}^{N_{\mathrm{iter}}}\sum_{i_{B}=1}^{N_{\mathrm{seg}}\left(j\right)}\left(N_{p\_\mathrm{slot}}\left(i_{B}\right)P_{\mathrm{seg}\_\mathrm{sel}}^{j,i_{B}}\left(\lambda ,R_{p\_B}^{n,i_{I},m}\right)\right)\right)+1\right)T_{\mathrm{slot}}$$

(22) $$T_{\mathrm{con}\_B}^{n,i,m}=T_{\mathrm{con}\_B}^{n,i_{I},m}=T_{\mathrm{con}}\left(\lambda ,R_{p\_B}^{n,i_{I},m}\right)=\sum_{i_{B}=1}^{N_{\mathrm{seg}}\left(N_{\mathrm{iter}}\right)}T_{\mathrm{con}\_\mathrm{seg}}^{i_{B}}\left(\lambda ,R_{p\_B}^{n,i_{I},m}\right)P_{\mathrm{seg}\_\mathrm{sel}}^{N_{\mathrm{iter}},i_{B}}\left(\lambda ,R_{p\_B}^{n,i_{I},m}\right)$$

From (14) and (15), the message progress $\beta_{B}^{n,i,m}$ and the $PDR_{B}^{n,i,m}$ on the *n*-th branch when ${\mathrm{Raley}}_{I}$ is in the $i_{I}$-th final segment can be derived as

(23) $$\beta_{B}^{n,i,m}=\beta_{B}^{n,i_{I},m}=\beta \left(\lambda ,R_{p\_B}^{n,i_{I},m}\right)=\sum_{i_{B}=1}^{N_{\mathrm{seg}}\left(N_{\mathrm{iter}}\right)}M_{i_{B}}P_{\mathrm{seg}\_\mathrm{sel}}^{N_{\mathrm{iter}},i_{B}}\left(\lambda ,R_{p\_B}^{n,i_{I},m}\right)$$

(24) $$PDR_{B}^{n,i,m}=PDR_{B}^{n,i_{I},m}=\;PDR\left(\lambda ,R_{p\_B}^{n,i_{I},m}\right)=\;1\;\;-\;\;\;\;\sum_{i_{B}=1}^{N_{\mathrm{seg}}\left(N_{\mathrm{iter}}\right)}\left(\left(\prod_{c=1}^{N_{\mathrm{recon}}}p_{\mathrm{col}\;\_\;\mathrm{con}}^{i_{B},c}\left(\lambda ,R_{p\;\_\;B}^{n,i_{I},m}\right)\right)P_{\mathrm{seg}\;\_\;\mathrm{sel}}^{N_{\mathrm{iter}},i_{B}}\left(\lambda ,R_{p\;\_\;B}^{n,i_{I},m}\right)\right)$$

As we know, ${\mathrm{Raley}}_{I}$ may exist on any branch, meanwhile ${\mathrm{Raley}}_{B}$ will be located on the branches except ${\mathrm{Br}}_{k}$ where message comes from. Moreover, the probability that the ${\mathrm{Raley}}_{I}$ lies on any branch is equal. Therefore, the average partition time $T_{\mathrm{part}\_B}$ and the average contention time $T_{\mathrm{con}\_B}$ in Branch Phase can be attained as

(25) $$T_{\mathrm{part}\_B}=\;\;\;\;\sum_{i=1}^{N_{\mathrm{seg}}\left(N_{\mathrm{iter}}\right)}\left(\frac{1}{N_{b}}\sum_{m=1}^{N_{b}}\left(\frac{1}{N_{b}-1}\sum_{n=1\&n\neq m}^{N_{b}}\;\;\;\;T_{\mathrm{part}\;\_\;B}^{n,i,m}\right)P_{\mathrm{seg}\;\_\;\mathrm{sel}}^{N_{\mathrm{iter}},i}\left(N_{b}\lambda ,\frac{R}{2}\right)\right)$$

(26) $$T_{\mathrm{con}\;\_\;B}=\;\;\;\;\sum_{i=1}^{N_{\mathrm{seg}}\left(N_{\mathrm{iter}}\right)}\left(\frac{1}{N_{b}}\sum_{m=1}^{N_{b}}\left(\frac{1}{N_{b}-1}\sum_{n=1\&n\neq m}^{N_{b}}\;\;\;\;T_{\mathrm{con}\;\_\;B}^{n,i,m}\right)P_{\mathrm{seg}\_\mathrm{sel}}^{N_{\mathrm{iter}},i}\left(N_{b}\lambda ,\frac{R}{2}\right)\right)$$

Message progress $\beta_{B}$ is defined as the average distance between ${\mathrm{Raley}}_{B}$ and C relative to *R*. From (14) and (15), $\beta_{B}$ and ${\mathrm{PDR}}_{B}$ can also be attained as

(27) $$\beta_{B}=\frac{1}{R}\sum_{i=1}^{N_{\mathrm{seg}}\left(N_{\mathrm{iter}}\right)}\left(\frac{1}{N_{b}}\sum_{m=1}^{N_{b}}\left(\frac{1}{N_{b}-1}\sum_{n=1\&n\neq m}^{N_{b}}\left(\beta_{B}^{n,i,m}R_{p\_B}^{n,i,m}+l_{C}^{i}\left(n==m\right)\right)\right)P_{\mathrm{seg}\_\mathrm{sel}}^{N_{\mathrm{iter}},i}\left(N_{b}\lambda ,\frac{R}{2}\right)\right)$$

and

(28) $$PDR_{B}=\;\;\;\;\sum_{i=1}^{N_{\mathrm{seg}}\left(N_{\mathrm{iter}}\right)}\left(\left(\frac{1}{N_{b}}\sum_{m=1}^{N_{b}}\left(\prod_{n=1\&n\neq m}^{N}\;\;\;\;PDR_{B}^{n,i,m}\right)\right)P_{\mathrm{seg}\_\mathrm{sel}}^{N_{\mathrm{iter}},i}\left(N_{b}\lambda ,\frac{R}{2}\right)\right)$$

#### 4.3.3. Metrics in Whole Procedure

Based on the previous results, the one-hop delay in each phase can be achieved from (5), and the delay $T_{\mathrm{two}\_\mathrm{hop}}$ in the whole relay-node selection on intersection is the summation of one-hop delay in two phases. β in the whole procedure can be derived as

(29) $$\beta =\left.\left(\beta_{B}R+\frac{R}{2}\right)/\frac{3R}{2}\right.=\left(2\beta_{B}+1\right)/3$$

From (6), we can get the message dissemination speed in the whole procedure. In addition, PDR in the whole procedure is

(30) $$PDR=PDR_{I}\times PDR_{B}$$

## 5. Numerical and Simulation Results

In the section, we will simulate our proposal and some prior works on a crossroad to validate the analytical models and to evaluate the performance of our proposal. The crossroad is a type of intersection with $N_{b}=4$ and $\left(\theta_{1},\theta_{2},\theta_{3},\theta_{4}\right)=\left(\pi /2,\pi ,3\pi /2,2\pi \right)$. These prior works include AMB in Ref. [18] and BPAB in Ref. [19]. $\left(N_{\mathrm{iter}},N_{\mathrm{part}}\right)$ in our proposal and BPAB are set as (2, 4) and (4, 2) according to [14] and [19], respectively, $N_{\max}=10$ for AMB and A = 2 for our proposal. To measure the gain benefited from the exponent-based partition method and mini-BBM [14], we also simulate an Iterative Partition with Equal Segment which has the same setting of $\left(N_{\mathrm{iter}},N_{\mathrm{part}}\right)$ with our proposal (referred to as IPES24) and the proposal without mini-BBM applied.

### 5.1. Simulation Environment

The simulation environment is VANET with MATLAB. Since the focus of these relay-node selection methods lies on link level, just 802.11p MAC layer is simulated in this paper. The major communication parameters are identical to those used in [13,14,19], listed in Table 2. Since most distance between adjacent intersections are in the range of (200,800) m, *R* is set as 400 m, a small value for *R* in VANET.

Each branch has a length of 700 m to ensure the simulation zone long enough for two hops. To assure nodes available in the partition range and evaluate the enhancement of our proposal in heavy traffic, the vehicle density λ for the simulation to compare the performance in Section 5.3 is set from 0.0025 to 3.0025 vehicle/meter at the interval of 0.2 vehicle/meter. Vehicles are distributed on every branch randomly, following Poisson distribution of the density λ. For simple simulation without loss of generality, the Hunter is located at the position R/2 far from C on the eastern branch in each simulation.

In the simulation, the value of the maximum speed $v_{\max}$ of vehicles is determined with the value of the distance between the adjacent vehicles to comply with the rule related to the safe inter-vehicle distance [26,27]. $v_{\max}$ can be given as

(31) $$v_{\max}=min\left(d_{\mathrm{inter}\_\mathrm{veh}},v_{\max\_\mathrm{rule}}\right)$$

where $d_{\mathrm{inter}\_\mathrm{veh}}$ is the average inter-vehicle distance, and $v_{\max\_\mathrm{rule}}$ represents the limit speed in a specific road scenario. The units of $v_{\max}$ and $d_{\mathrm{inter}\_\mathrm{veh}}$ are km/h and m, respectively. Each vehicle chooses a random speed following a uniform distribution in $\left(\frac{1}{2}v_{\max},v_{\max}\right)$ at the beginning of the simulation and keeps the chose speed during the simulation. Lane change and overtaking are not modeled for vehicle movement. From the simulation results, the duration for the message going through the intersection range is less than 3 ms. Moreover, the minor impact of node mobility on the relay-node selection has been proved in Ref. [28,29]. Hence, the above assumptions about the vehicle movement and the adoption of MATLAB are acceptable.

The arrival rate of messages is set to 2 EMs/s, $N_{\mathrm{recon}}=3$ for the validation of the PDR model to separate the curves and $N_{\mathrm{recon}}$ for others. $C_{W(1)}$ is chose as 1 for AMB because of a few candidates in the CTB contention, and 4 for others. $T_{m\_\mathrm{slot}}$ in mini-BBM and $T_{\mathrm{inter}}$ in CTB contention are selected as $T_{\mathrm{inter}}/3$ and $T_{\mathrm{inter}}/2$ to reduce the partition latency in sparse vehicles and the contention latency in heavy traffic, meanwhile to avoid the spurious forwarding [30].

We performed 20,000 repetitions of Monte Carlo simulation [31,32] for PDR results and 1000 repetitions for other results to get statistical significance. These outcomes are averaged to produce the graphs presented in this section with 95% confidence intervals. The confidence intervals are marked with the error bars in the plots.

### 5.2. Validations of Analytical Model

**Figure 3:**
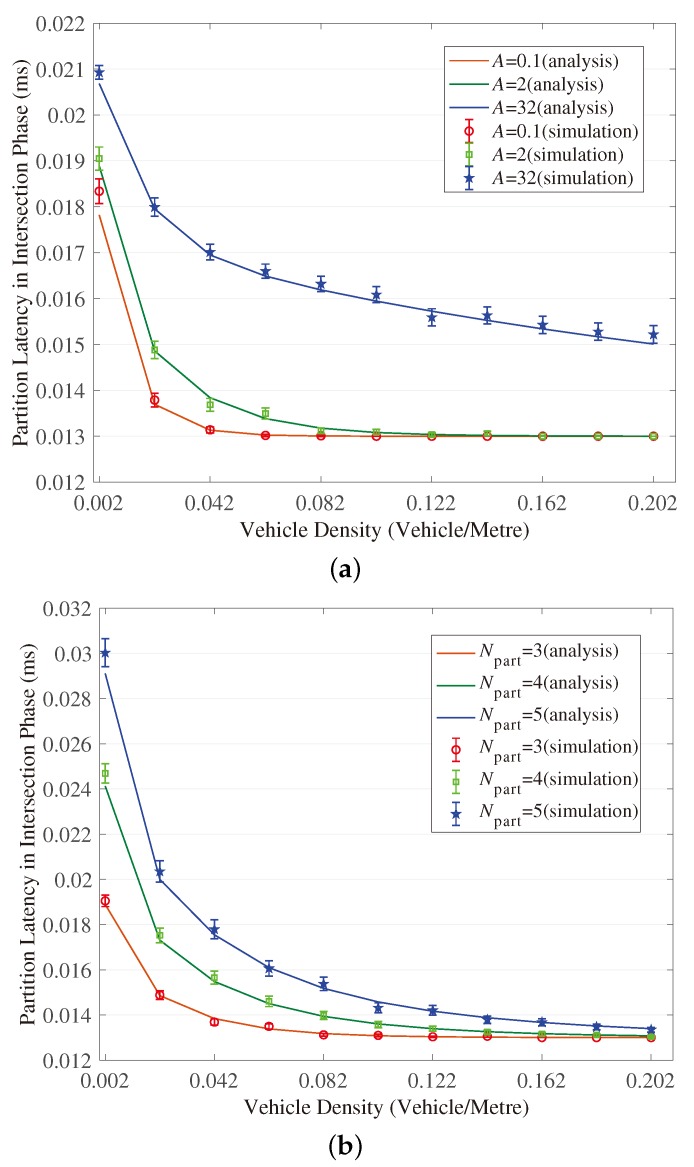

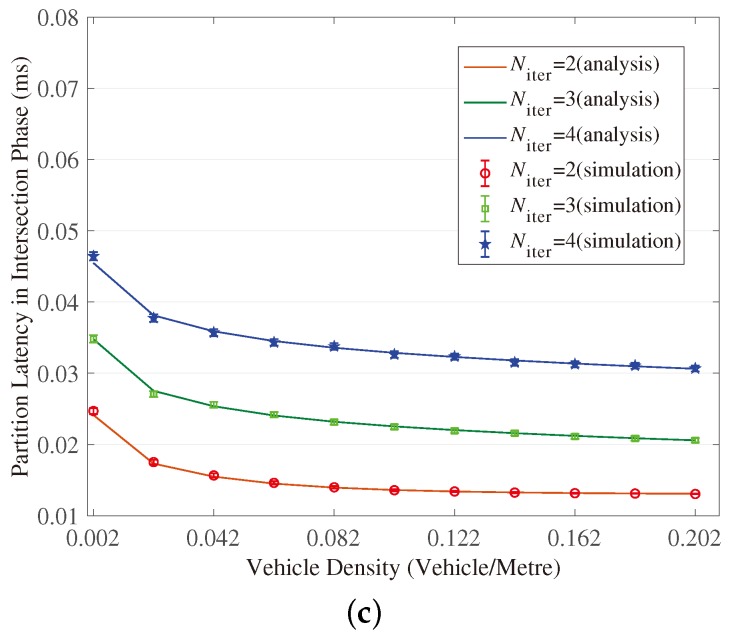
Validation of the model for partition latency in Intersection Phase. (**a**) When $\left(N_{\mathrm{iter}},N_{\mathrm{part}}\right)$ = (2, 3); (**b**) When $\left(N_{\mathrm{iter}},A\right)=\left(2,2\right)$; (**c**) When $\left(N_{\mathrm{part}},A\right)=\left(4,2\right)$.

**Figure 4:**
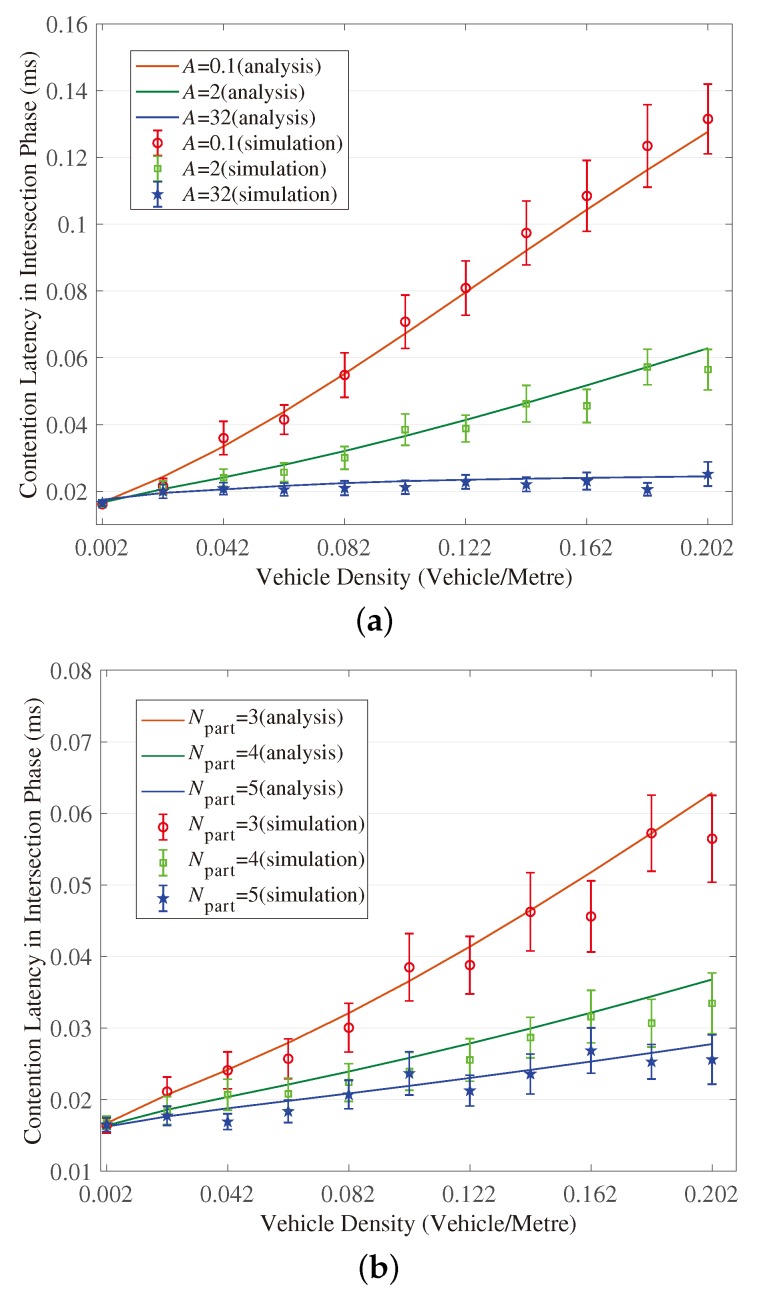

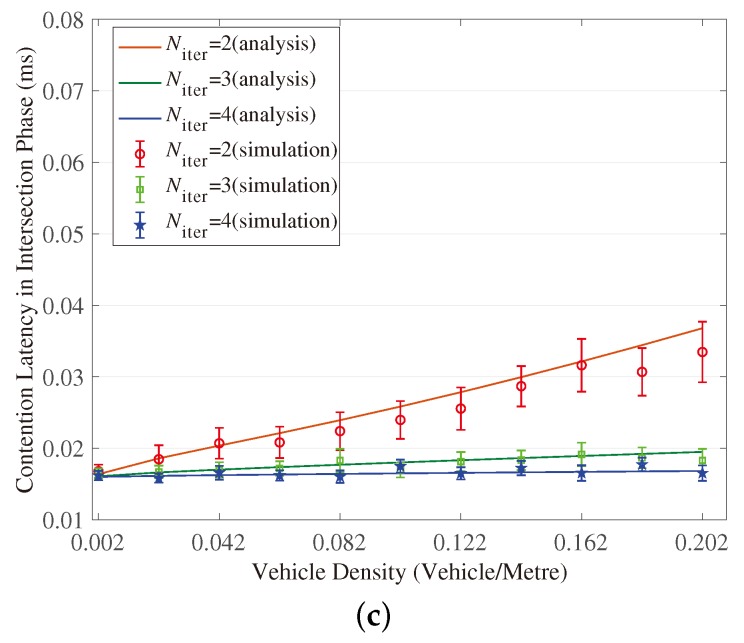
Validation of the model for contention latency in Intersection Phase. (**a**) When $\left(N_{\mathrm{iter}},N_{\mathrm{part}}\right)$ = (2, 3); (**b**) When $\left(N_{\mathrm{iter}},A\right)=\left(2,2\right)$; (**c**) When $\left(N_{\mathrm{part}},A\right)=\left(4,2\right)$.

**Figure 5:**
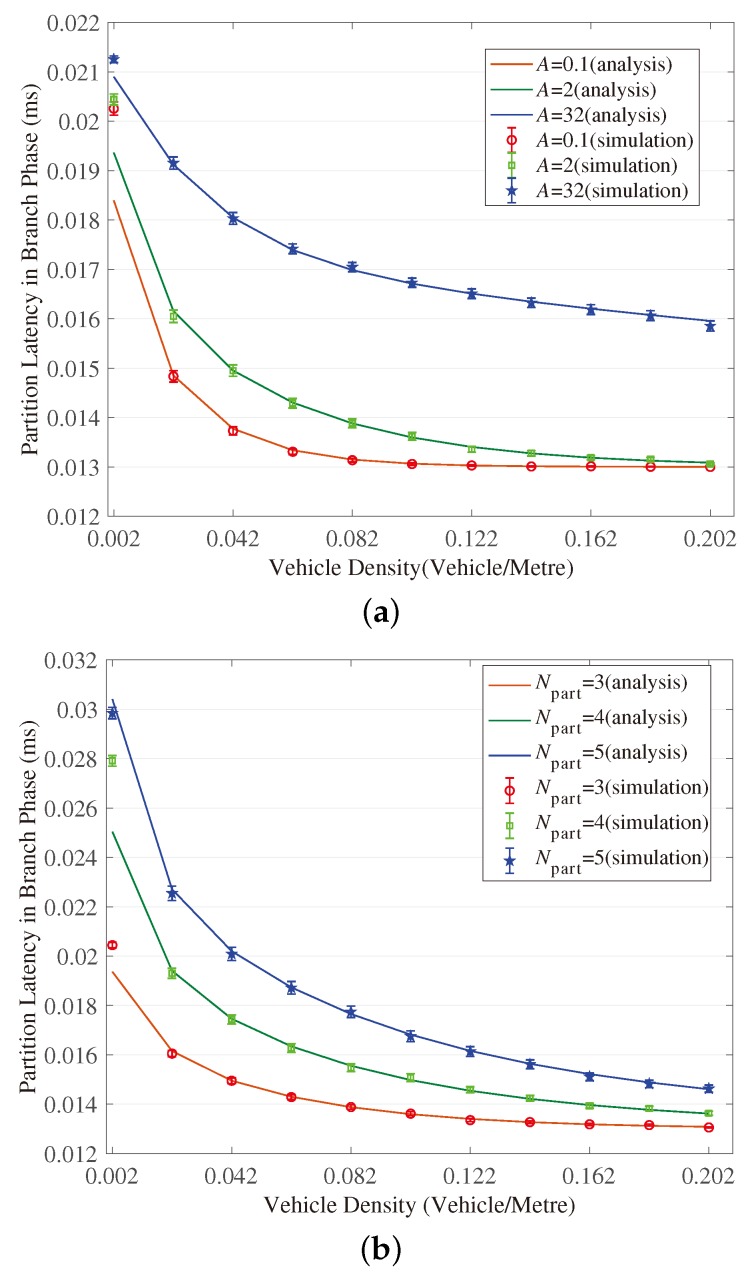

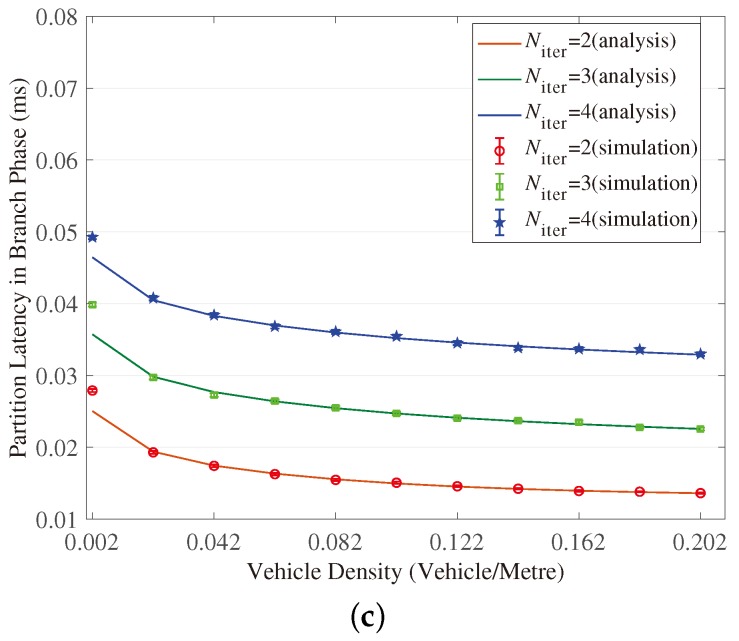
Validation of the model for partition latency in Branch Phase. (**a**) When $\left(N_{\mathrm{iter}},N_{\mathrm{part}}\right)=\left(2,3\right)$; (**b**) When $\left(N_{\mathrm{iter}},A\right)=\left(2,2\right)$; (**c**) When $\left(N_{\mathrm{part}},A\right)=\left(4,2\right)$.

**Figure 6:**
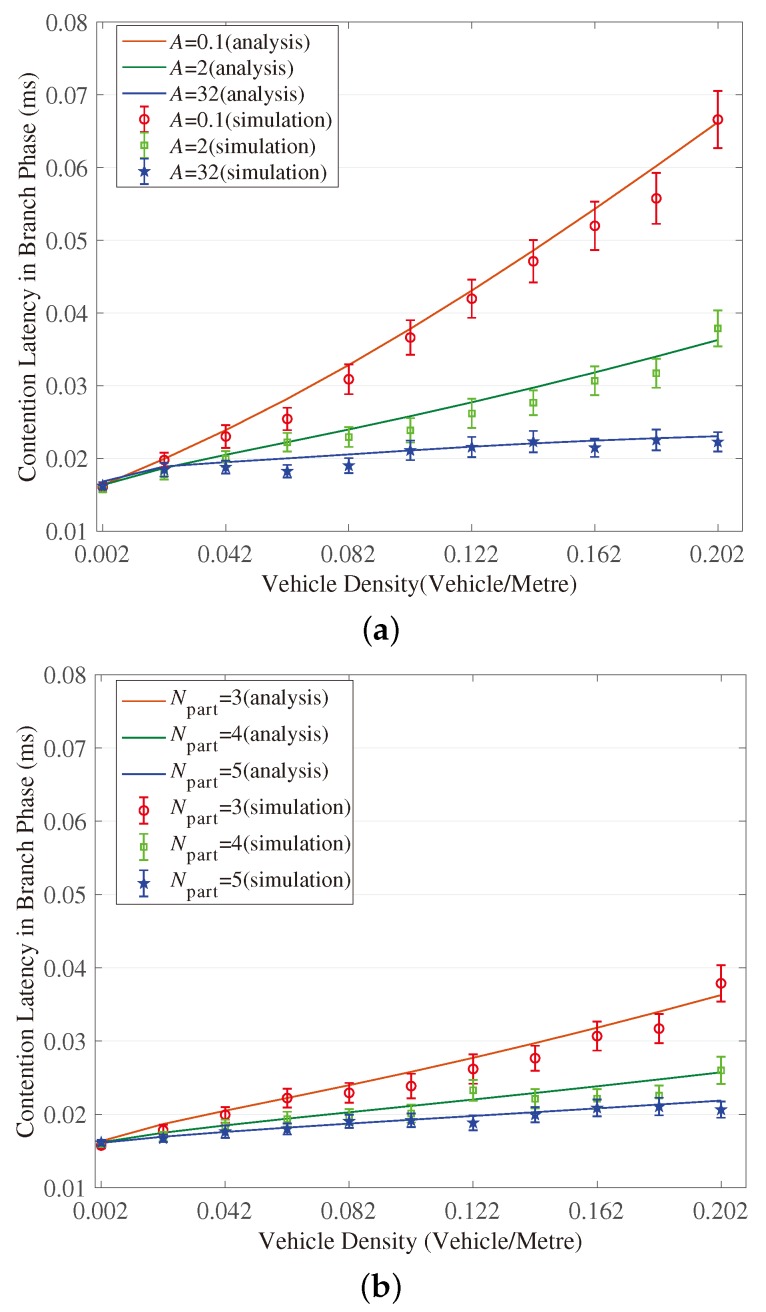

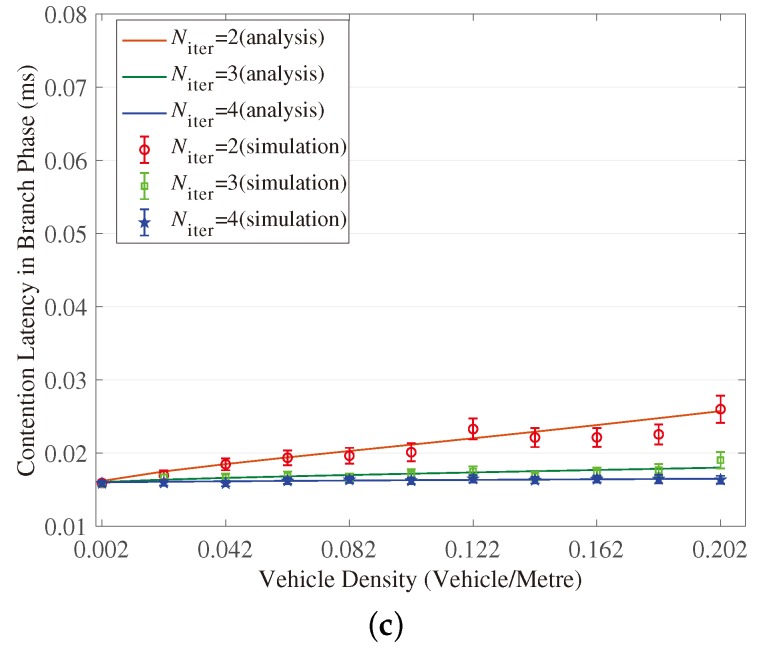
Validation of the model for contention latency in Branch Phase. (**a**) When $\left(N_{\mathrm{iter}},N_{\mathrm{part}}\right)$ = (2, 3); (**b**) When $\left(N_{\mathrm{iter}},A\right)=\left(2,2\right)$; (**c**) When $\left(N_{\mathrm{part}},A\right)=\left(4,2\right)$.

**Figure 7:**
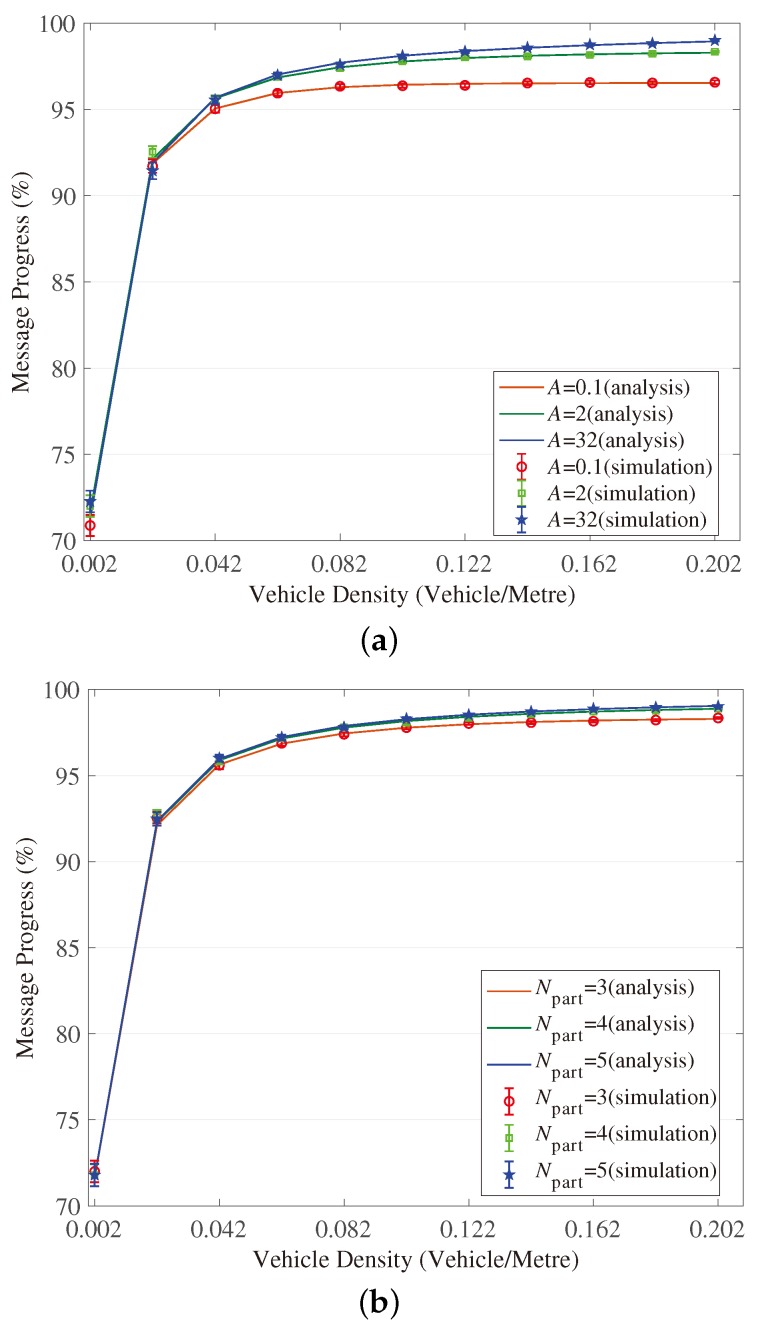

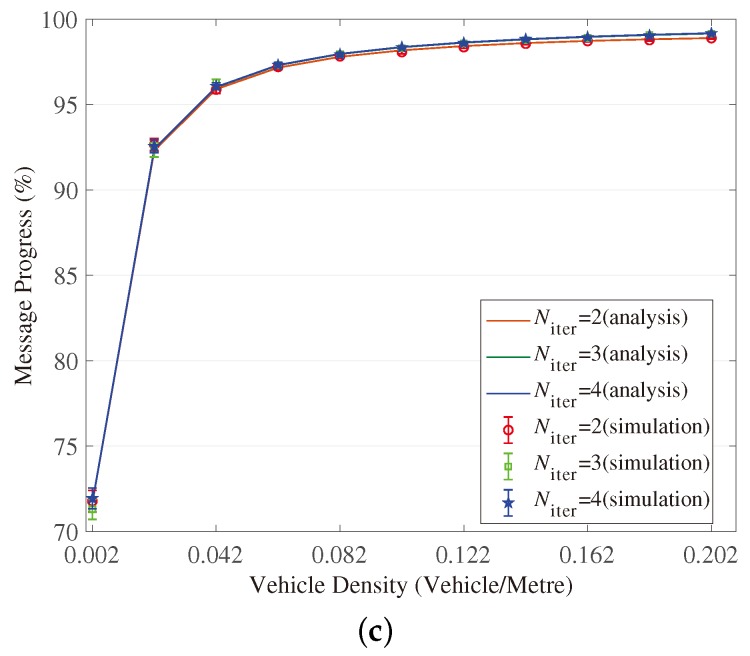
Validation of the model for message progress in Branch Phase. (**a**) When $\left(N_{\mathrm{iter}},N_{\mathrm{part}}\right)=\left(2,3\right)$; (**b**) When $\left(N_{\mathrm{iter}},A\right)=\left(2,2\right)$; (**c**) When $\left(N_{\mathrm{part}},A\right)=\left(4,2\right)$.

**Figure 8:**
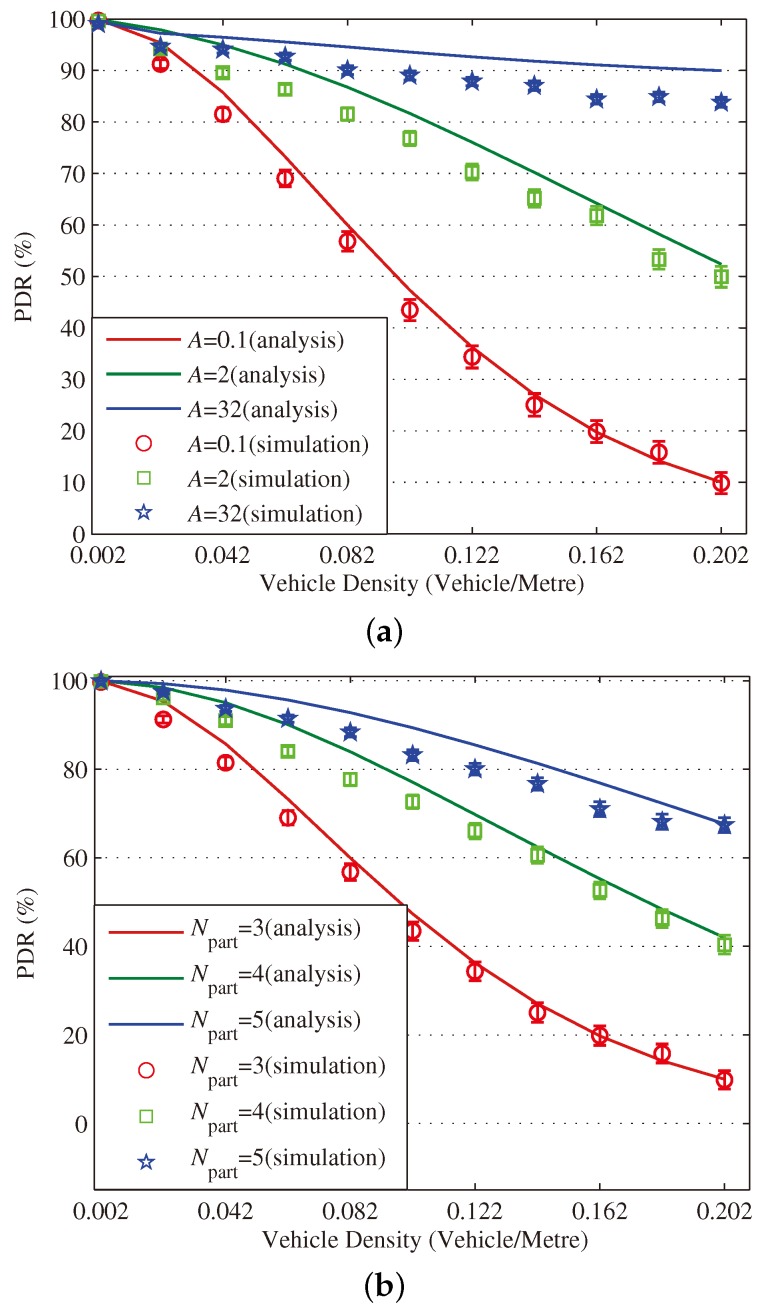

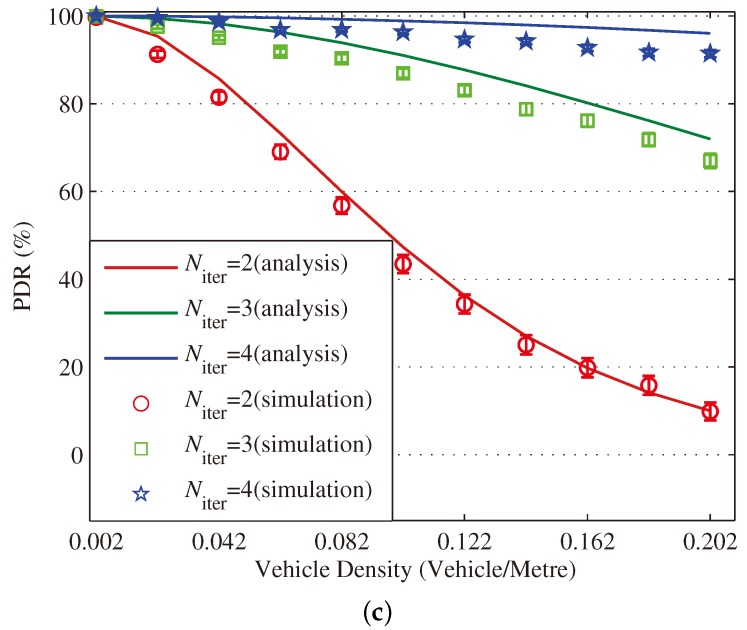
Validation of the analytical model for PDR. (**a**) When $\left(N_{\mathrm{iter}},N_{\mathrm{part}}\right)=\left(2,3\right)$; (**b**) When $\left(N_{\mathrm{iter}},A\right)=\left(2,0.1\right)$; (**c**) When $\left(N_{\mathrm{part}},A\right)=\left(3,0.1\right)$.

Figure 3, Figure 4, Figure 5, Figure 6, Figure 7 and Figure 8 show the comparison of the results of analytical models (lines) and the simulation (symbols). These results are a function of vehicle density with varied $N_{\mathrm{part}}$, $N_{\mathrm{iter}}$ and *A*. As can be seen from these figures, the analytical predictions coincide with simulation results well, showing the validity of the obtained analytical expressions.

From these figures, some new interesting observations can be got as follows: (1) With one of the three parameters ($N_{\mathrm{part}}$, $N_{\mathrm{iter}}$ and *A*) decreasing, the partition latency is reduced. In addition, the gain of the partition latency benefited from the decreasing of the three parameters falls as the parameter becomes a smaller value. The similar tendency is also observed for the contention latency, message progress and PDR with the three parameters increasing. (2) The partition latency approaches a constant value when the traffic becomes heavier. This is because when the vehicle density rises over a particular value, at least one node exists in the final segment near the optimal position, then the number of B spent in the partition phase is fixed as $N_{\mathrm{iter}}-1$. (3) We also find that the bigger values have the three parameters, the smaller the contention latency varies with density, shown as the lowest curve in Figure 4 and Figure 6. The observation is because the bigger values of the parameters can result in a thinner final segment. (4) Increasing these parameters can improve the contention latency but prolong the partition latency, and the influence is different at different vehicle densities. It is confirmed again that there are some optimal values for these three parameters to get a maximum message speed.

### 5.3. Evaluations of Performance

**Figure 9:**
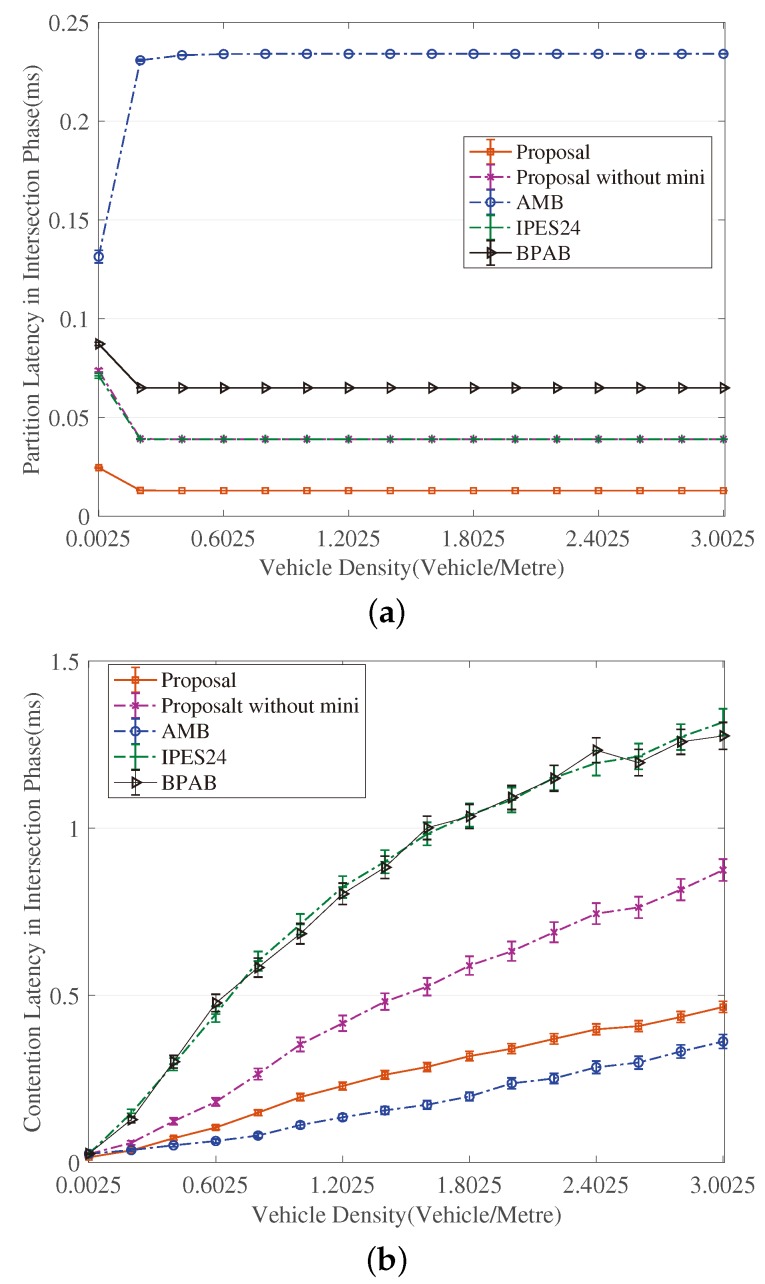
Comparison of Latency in Intersection Phase. (**a**) Comparison of partition latency; (**b**) Comparison of contention latency.

**Figure 10:**
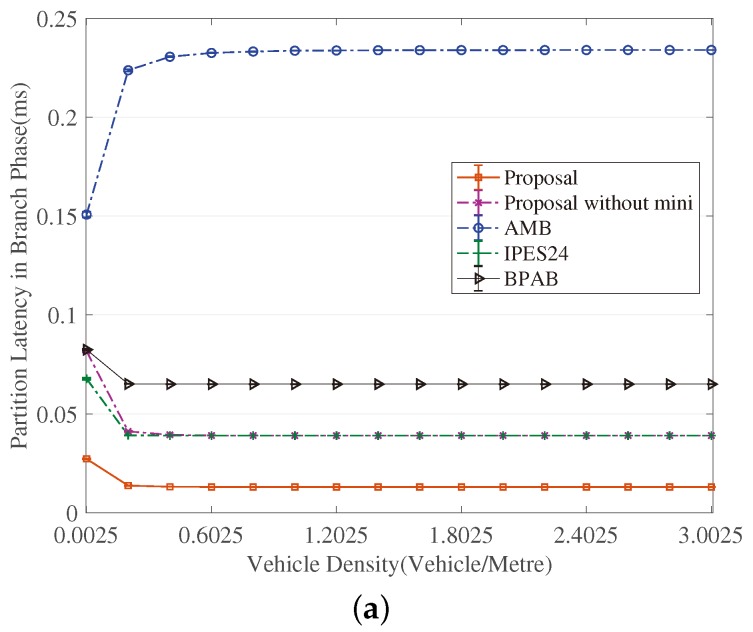

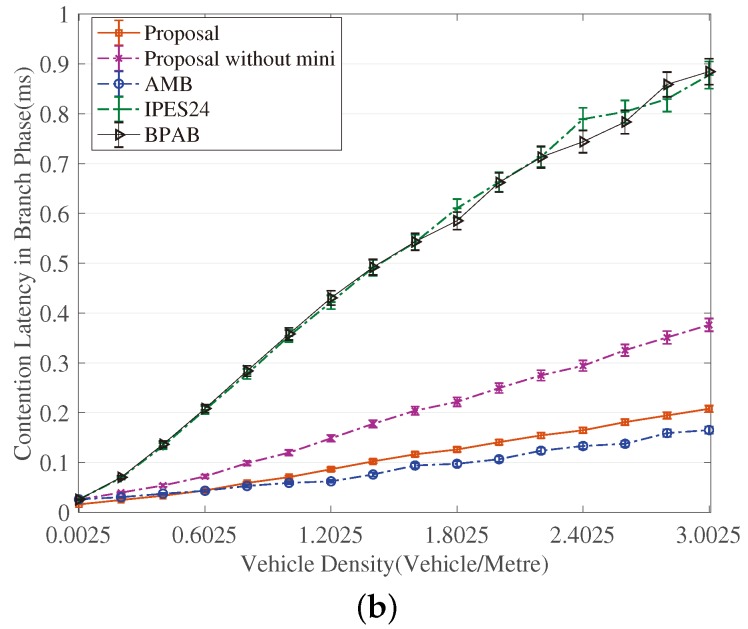
Comparison of Latency in Branch Phase. (**a**) Comparison of partition latency. (**b**) Comparison of contention latency.

Figure 9 and Figure 10 reveal that the exponent partition mechanism is advantageous to the partition latency within a limited value (seen from the curves of ‘’Proposal without mini” in Figure 9a and Figure 10a), meanwhile is favorable of a significant gain in terms of the contention latency compared to BPAB and IPES24 (seen from Figure 9b and Figure 10b). Although at high vehicle densities (e.g., 3.0025 vehicles/meter), the gain in partition latency cannot make up the gap in contention latency compared to AMB, the mini-BBM mechanism benefits our proposal to defeat AMB in terms of the sum of partition latency and contention latency.

**Figure 11:**
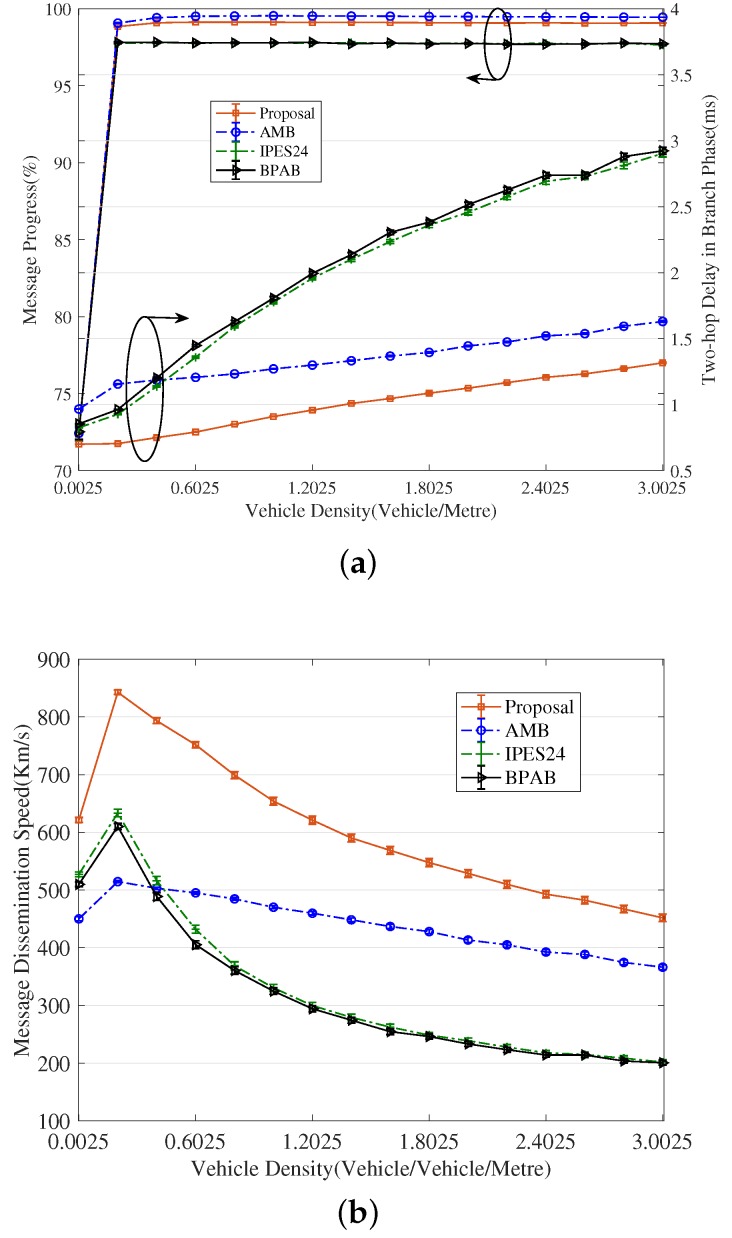
Comparison of efficient performance in whole procedure. (**a**) Comparison of two-hop delay and message progress; (**b**) Comparison of dissemination speed.

As a result, shown in Figure 11a, our proposal achieves more than 18.10%, 15.26% and 19.24% lower two-hop delay than that of BPAB, IPES24 and AMB. In particular in heavy traffic, the enhancement is more significant. Additionally, our proposal attains the second-best performance in terms of message progress, which is only 0.37% worse than the best performing AMB. Consequently, it can be observed in Figure 11b that the message dissemination speed achieved by our proposal outperforms that of BPAB, IPES24 and AMB by at least 21.89%, 17.87% and 23.35%. The improvement even goes up to 125.23%, 123.87% compared to BPAB and IPES24 at the high vehicle density of 3.0025 vehicles/meter, and 63.78% compared to AMB at the relatively low vehicle density of 0.2025 vehicles/meter. It is also observed from Figure 11b that, at the heavy vehicle density, the performance of BPAB and IPES24 in terms of message dissemination speed drops gently, not as drastically as expected. The reason is that when the re-attempt number of CTB contention is over the pre-selected number $N_{\mathrm{recon}}$, the performance of this case in terms of message dissemination speed does not need to be evaluated, and the contention latency in average one-hop delay is decided by the duration of $N_{\mathrm{recon}}$ contentions in heavy traffic.

**Figure 12:**
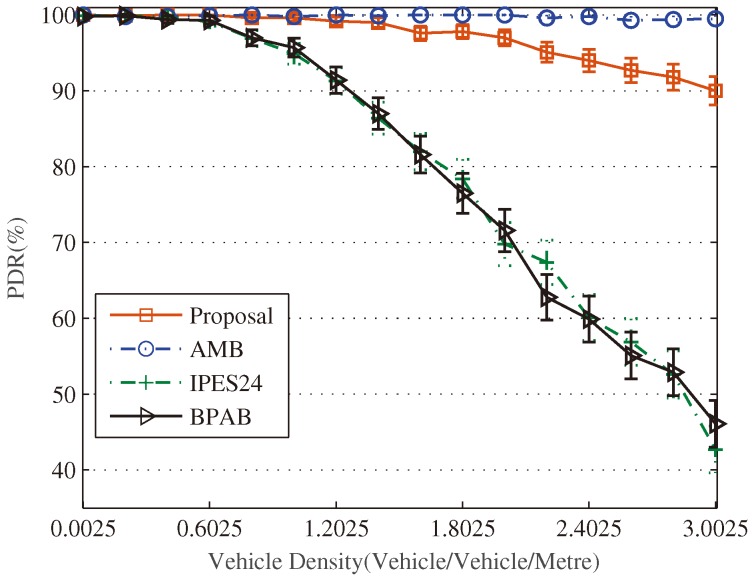
Comparison of PDR.

Figure 12 demonstrates that our proposal presents the second-best performance in terms of PDR, which is over 90% stably. Although our proposal performs worse than the best performing AMB, it can be promoted by choosing a bigger A in dense networks. It is also observed that the performance of BPAB and IPES24 in terms of PDR degrades sharply as the vehicle density increases.

From the results in Figure 9, Figure 10, Figure 11 and Figure 12 and the above analysis, some conclusions and suggestions can be given as follows: (1) In traffic jams, effective and real-time message dissemination is highly needed. However, the relay-node selection is more likely to fail due to the high collision rate caused by the high number of vehicles, and the performance will deteriorate in terms of message dissemination speed and PDR. In this sense, our proposal provides an effective solution by employing the design of exponent-based partition. It generates thinner final segments and thus improves message dissemination speed and PDR in the dense traffic. (2) From Figure 11b, it is clear to observe that all approaches perform the best message dissemination speed at the density of 0.2025 vehicle/meter. It demonstrates that the relay-node selection method based on the distance has an optimal vehicle density at which the method performs the best in terms of the message dissemination speed. Thus, when considering the real-time dissemination of the message, to select the branch that has the vehicle density closest the optimal value is a good suggestion in the routing design [33,34,35,36,37,38].

## 6. Conclusions

In this paper, after building a model for a general intersection with any number of branches and any angles between branches, we investigated a general relay-node selection method based on exponent-based partition. Several mechanisms are combined to improve the performance, including: the design of the minimum partition range and mini-BBM mechanism. Compared with the prior methods, our proposal gains remarkable improvement in efficiency and reliability. In addition, based on the mathematical description of the general intersection, we explore the analytical model for performance in terms of both the message dissemination speed and PDR. Our work focuses on modeling the partition latency, contention latency, message progress and PDR, which the exponent partition mechanism will affect. These models account for the adaptation of the exponential back-off timer. The results of computer simulation justify the accuracy of these models and the improvement of our proposal.

Some interesting observations in the paper bring up some instructive ideas, such as the branch selection based on the optimal vehicle density in the route problem, and the parameter optimization according to the communication range and the vehicle density. In the future, we will further our work on the design of the relay-node selection on the curve road, a typical road structure, and an adaptive relay-node selection scheme aided with NS-3 and mobility generators such as Bonnmotion including more-realistic traffic scenarios.

## Figures and Tables

**Table 1:** Summary of notations.

Notations	Descriptions
B	black burst
*R*	communication range
$N_{\mathrm{part}}$	number of partitions in each iteration
$N_{\mathrm{iter}}$	number of iterations
*A*	compression coefficient
$N_{b}$	number of branches
C	center of intersection
${\mathrm{Br}}_{n}$	the *n*-th branch
$\theta_{n}$	angle between the *n*-th branch and positive axis
H	hunter
${\mathrm{Relay}}_{I}$	relay node in Intersection Phase
${\mathrm{Relay}}_{B}$	relay node in Branch Phase
*r*	radius of intersection range
*m*	index of the branch ${\mathrm{Relay}}_{I}$
$l_{B}^{n}$	coverage of H on the *n*-th branch
${\mathrm{Relay}}_{B}^{n}$	relay node in Branch Phase on the *n*-th branch
$P_{\mathrm{opt}}^{n}$	optimal oint on the *n*-th branch
$R_{p}$	partition range
$D_{\bar{A,B}}$	range between the points of A and B
$T_{d}$	one-hop delay
$T_{\mathrm{init}}$	initial latency
$T_{\mathrm{part}}$	average partition latency
$T_{\mathrm{con}}$	average contention latency
$T_{\mathrm{data}}$	data transmission latency
*v*	message dissemination speed
$\hat{T_{d}}$	delay in the whole procedure
β	average one-hop message progress
$R_{\max}$	maximal distance of message dissemination along the road in the whole procedure
PDR	packet delivery ratio
$\hat{\lambda }$	equivalent node density
$P_{\mathrm{seg}\_\mathrm{sel}}^{j,i}$	probability of the selection of the *i*-th segment in the *j*-th iteration
$\mu_{\mathrm{seg}\_\mathrm{bro}}^{j,i}$	average vehicle numbers in other segments in the message dissemination direction when the *i*-th segment is selected in the *j*-th iteration
$\mu_{\mathrm{seg}}^{j,i}$	the average vehicle numbers in the *i*-th segment of the *j*-th iteration
$N_{P\_\mathrm{slot}}$	number of time slots spent when a segment is selected
$T_{\mathrm{slot}}$	duration of a time slot
$N_{\mathrm{seg}}\left(j\right)$	number of segments in the *j*-th iteration
$p_{\mathrm{suc}\_\mathrm{con}}^{i,c}$	single probability of the success case in the *c*-th contention of the *i*-th final segment
$p_{\mathrm{col}\_\mathrm{con}}^{i,c}$	single probability of the collision case in the *c*-th contention of the *i*-th final segment
$p_{c}$	probability of the selection of a back-off timer in the *c*-th contention
$C_{w}\left(c\right)$	maximal number of back-off timers in the *c*-th contention
$p_{\mathrm{suc}}^{i,c}$	whole success probability after *c* contentions in the *i*-th final segment
$T_{\mathrm{con}\_\mathrm{seg}}^{i}$	contention latency of the *i*-th segment
$T_{\mathrm{con}\_\mathrm{si}}\left(c\right)$	durations spent in the collision case in the *c*-th contention
$T_{\mathrm{suc}\_\mathrm{si}}\left(c\right)$	durations spent in the success case in the *c*-th contention
$N_{\mathrm{recon}}$	number of the contention re-attempt
$T_{\mathrm{part}\_I}$	partition latency in Intersection Phase
$T_{\mathrm{con}\_I}$	contention latency in Intersection Phase
$\beta_{I}$	message progress in Intersection Phase
$PDR_{I}$	PDR in Intersection Phase
$R_{p\_B}^{n,i,m}$	partition range on the *n*-th branch in Branch Phase when ${\mathrm{Raley}}_{I}$ is in the *i*-th final segment on the *m*-th branch
$T_{\mathrm{part}\_B}^{n,i,m}$	partition latency on the *n*-th branch in Branch Phase when ${\mathrm{Raley}}_{I}$ is in the *i*-th final segment on the *m*-th branch
$T_{\mathrm{con}\_B}^{n,i,m}$	contention latency on the *n*-th branch in Branch Phase when ${\mathrm{Raley}}_{I}$ is in the *i*-th final segment on the *m*-th branch
$\beta_{B}^{n,i,m}$	message progress on the *n*-th branch when ${\mathrm{Raley}}_{I}$ is in the $i_{I}$-th final segment on the *m*-th branch
$PDR_{B}^{n,i,m}$	PDR on the *n*-th branch when ${\mathrm{Raley}}_{I}$ is in the $i_{I}$-th final segment on the *m*-th branch
$T_{\mathrm{part}\_B}$	partition latency in Branch Phase
$T_{\mathrm{con}\_B}$	contention latency in Branch Phase
$\beta_{B}$	message progress in Branch Phase
$PDR_{B}$	PDR in Branch Phase
$v_{\max}$	maximum speed of vehicles
$d_{\mathrm{inter}\_\mathrm{veh}}$	average inter-vehicle distance
$v_{\max\_\mathrm{rule}}$	limit speed in a specific road scenario

**Table 2:** Major communication parameters.

Parameters	Default Values
Bit Rate	18 Mbps
Message Packet Size	500 Bytes
RTB Packet Size	20 Bytes
CTB Packet Size	14 Bytes
Slot Time	13 μs
DIFS	58 μs
SIFS	32 μs
Transceiver’s Switching Time	1 μs
Communication Range	400 m
Confidence Interval	95%

## Abbreviations

The following abbreviations are used in this manuscript:

VNET	Vehicular NETwork
NLOS	Non-Line-Of-Sight
PDR	Packet Delivery Ratio
VANET	Vehicular Ad hoc NETworks
LTE eV2X	Long-Term Evolution-based enhanced Vehicle-to-Everything
GIS	Geographic Information System
GPSR	Greedy Perimeter Stateless Routing
PMBP	Position-based Multi-hop Broadcast Protocol
3P3B	Trinary Partitioned Black-Burst-based Broadcast protocol
UMB	Urban Multi-hop Broadcast protocol
AMB	Ad hoc Multi-hop Broadcast
BPAB	Binary-Partition-Assisted Broadcast protocol
EPBP	Exponent-based Partitioning Broadcast Protocol
CTB	Clear-To-Broadcasting
RTB	Request-To-Broadcasting
mini-BBM	mini-Black-Burst-assisted Mechanism

## Appendix A. Proof of the Partition Range in Branch Phase

Assume message comes from the *k*-th branch, and ${\mathrm{Relay}}_{I}$ lies on the *m*-th branch. One ${\mathrm{Relay}}_{B}$ is expected to be selected on the *n*-th branch. It is worth pointing out that m≠n. Denote the distance between ${\mathrm{Relay}}_{I}$ and the center of intersection (C) as $l_{C}$, and the distance between the optimal position $P_{\mathrm{opt}}$ on the *n*-th branch and C as $l_{B}^{n}$. The relationship between $l_{C}$ and $l_{B}^{n}$ can be presented as

(A1) $$\left\{\begin{array}{ccc} l_{B}^{n}\;\;\;\;\;\; & =R+l_{C}, & \mathrm{when}\;n=m\\ R^{2}\;\;\;\;\;\; & ={l_{C}}^{2}+{\left(l_{B}^{n}\right)}^{2}-2l_{C}l_{B}^{n}\cos\left(\theta_{n}-\theta_{m}\right), & \mathrm{when}\;n\neq m \end{array}\right.$$

The second sub-equation in the above equation can be represented as

(A2) $${\left(l_{B}^{n}\right)}^{2}-2l_{C}\cos\left(\theta_{n}-\theta_{m}\right)l_{B}^{n}+\left({l_{C}}^{2}-R^{2}\right)=0$$

Since $l_{C}\leq R/2$, then

(A3) $$R^{2}\geq \frac{R^{2}}{4}\geq {l_{C}}^{2}\geq \left(1-\cos^{2}\left(\theta_{n}-\theta_{m}\right)\right){l_{C}}^{2}$$

So

(A4) $${\left(2l_{l}\cos\left(\theta_{n}-\theta_{m}\right)\right)}^{2}-4\left({l_{C}}^{2}-R^{2}\right)\geq 0$$

From the above analysis, we can derive that $l_{B}^{n}$ obtained from (A2) has a real value. Moreover, $l_{B}^{n}\geq 0$. Then $l_{B}^{n}$ can be presented as

(A5) $$l_{B}^{n}=\left\{\begin{array}{l} R+l_{C},\mathrm{when}\;n=m\\ l_{C}\cos\left(\theta_{n}-\theta_{m}\right)+\sqrt{R^{2}-{l_{C}}^{2}\sin^{2}\left(\theta_{n}-\theta_{m}\right)},\mathrm{when}\;n\neq m \end{array}\right.$$

Since ${\mathrm{Relay}}_{B}$ is expected to be closer to the optimal position than ${\mathrm{Relay}}_{I}$, the partition range for ${\mathrm{Relay}}_{B}$ is the range between $P_{\mathrm{opt}}$ and ${\mathrm{Relay}}_{I}$ when ${\mathrm{Relay}}_{B}$ and ${\mathrm{Relay}}_{I}$ on the same branch (i.e., m=n). Therefore, the partition range $R_{p\_B}^{n,i,m}$ in the branch phase on the *n*-th branch when ${\mathrm{Relay}}_{I}$ lies in the *i*-th final segment on the *m*-th branch is

(A6) $$R_{p\_B}^{n,i,m}=\left\{\begin{array}{l} R,\mathrm{when}\;n=m\\ l_{C}^{i}\cos\left(\theta_{n}-\theta_{m}\right)\;\;+\;\;\sqrt{R^{2}-(l_{C}^{i}{)}^{2}\sin^{2}\left(\theta_{n}-\theta_{m}\right)},\mathrm{when}\;n\neq m \end{array}\right.$$

The expected location of the relay node is the point in the middle of the final segment. Thus, when ${\mathrm{Relay}}_{I}$ is in the *i*-th final segment, the distance $l_{C}^{i}$ can be expected as

(A7) $$l_{C}^{i}=\left(\sum_{b=1}^{i-1}\left(W_{\mathrm{seg}(N_{\mathrm{iter}},b)}\right)+\frac{1}{2}W_{\mathrm{seg}(N_{\mathrm{iter}},i)}\right)\times R$$

where $W_{\mathrm{seg}}\left(N_{\mathrm{iter}},i\right)$ can be obtained from (1).
